# Forms of spirituality and their associations with conspiratorial thinking in Polish young adults

**DOI:** 10.3389/fpsyt.2026.1768454

**Published:** 2026-02-27

**Authors:** Patryk Główczyński, Paweł Dębski, Karina Badura-Brzoza

**Affiliations:** Clinical Department of Psychiatry, Faculty of Medical Sciences in Zabrze, Medical University of Silesia, Katowice, Poland

**Keywords:** conspiracy beliefs, conspiratorial thinking, nonreligious forms of spiritual practice, spirituality, young adults

## Abstract

**Background:**

Conspiracy beliefs are increasingly recognized as relevant to mental health, treatment adherence, and health-related behaviors, particularly among young adults. At the same time, patterns of spirituality in this group are shifting from institutional religiosity toward more individualized forms of spiritual practice. While spirituality is often considered a protective resource, less is known about how different forms of spirituality - religious, non-religious, and syncretic - are associated with conspiratorial thinking in young adults living in rapidly changing sociocultural contexts.

**Methods:**

We conducted a cross-sectional study among 1,100 young adults (aged 18–25 years) in Poland who were not undergoing psychiatric or psychological treatment. Based on self-declared worldview and engagement in Nonreligious Forms of Spiritual Practice (NFSP)—defined as spiritual practices pursued outside institutional religion—participants were classified into four groups: Catholics, Catholics engaging in NFSP (syncretic spirituality - Catholics Plus), atheists, and atheists engaging in NFSP - Atheists Plus. Spirituality was assessed using the Interfaith Spirituality Scale (ISS), and conspiratorial thinking with the Generic Conspiracist Beliefs Scale (GCBS). Group differences and within-group correlations between ISS domains and GCBS scores were analyzed using nonparametric methods.

**Results:**

Participants engaging in NFSP—both religious and non-religious—displayed higher levels of conspiratorial thinking than their counterparts who did not engage in such practices. The highest GCBS scores were observed in the syncretic group (Catholics engaging in NFSP), followed by atheists engaging in NFSP, Catholics, and atheists. Among Catholics, higher spirituality—particularly in the domain of asceticism and moral self-regulation—was negatively associated with conspiratorial thinking. Among atheists, ascetic spirituality also showed a protective association, whereas domains reflecting intuitive or transcendent connection were positively related to selected conspiracy belief dimensions. In both NFSP groups, higher spirituality—especially in meditation- and experience-oriented domains—was consistently associated with stronger conspiratorial beliefs.

**Conclusions:**

The findings suggest that spirituality is not a homogeneous construct in relation to conspiratorial thinking. Institutionally embedded, norm-regulated spirituality may be associated with lower endorsement of conspiracy beliefs, whereas individualized, non-religious forms of spiritual practice appear to co-occur with higher susceptibility to conspiratorial narratives among Polish young adults. These relationships should be interpreted as associational rather than causal and highlight the importance of considering the form and epistemic orientation of spirituality in research on conspiracy beliefs.

## 1 Introduction

### 1.1 Conspiracy beliefs and their relevance to mental health

In recent years, conspiracy beliefs (also referred to as conspiracism or conspiracy thinking) have become an increasingly frequent subject of research in psychology and public health, owing to their association with mental well-being, psychosocial functioning, and adherence to therapeutic recommendations (1–3). These beliefs are defined as explanations of significant events based on the assumption of secret actions by influential individuals or groups. Contrary to common misconceptions, conspiracy beliefs are not limited to individuals with psychotic disorders; they are widespread in the general population, including among adolescents and young adults (4, 5). Such beliefs may serve as coping strategies in situations characterized by uncertainty, lack of control, or stress caused by rapid social change. However, they often lead to increased mistrust, social withdrawal, and susceptibility to misinformation (6–8). The rapid development of digital media has further amplified the tendency toward conspiratorial thinking. This phenomenon may be particularly relevant among young adults, who exhibit high levels of online activity and are still in the process of identity formation (9).

### 1.2 Spiritual characteristics of young adults

In psychological and sociological research, the terms religiosity and spirituality are often used interchangeably, although they refer to partially distinct dimensions of human experience. Religiosity denotes affiliation with an institutionalized religious tradition, including shared doctrines, rituals, and community membership. Spirituality, by contrast, refers to a broader and more individualized orientation toward transcendence, meaning-making, and inner transformation, which may—but does not have to—be embedded in organized religion (10, 11). Contemporary definitions of spirituality usually emphasize three interrelated dimensions: transcendence, understood as reference to realities beyond everyday experience; the search for meaning, centered on ultimate values and life purpose; and personal transformation, involving identity development and moral self-regulation (10). Within this framework, different forms of spirituality can be distinguished. Religious spirituality refers to spiritual experience embedded in institutional religious traditions. Non-religious spirituality denotes individualized forms of spiritual engagement that develop outside formal religious structures. In the present study, we further operationalize this domain through the concept of Nonreligious Forms of Spiritual Practice (NFSP), defined as spiritual practices pursued outside institutional religion and shaped primarily by personal conviction, subjective experience, and informal cultural transmission. The term is used in this study as an operational category rather than a theoretical construct. NFSP include activities such as meditation practiced outside religious frameworks, astrology, divination, manifestation techniques, and other practices oriented toward personal meaning-making, intuitive insight, or metaphysical interpretations of reality. Importantly, the term is used descriptively rather than evaluatively, and does not imply that these practices are intrinsically irrational or pathological. Rather, it serves to distinguish institutionalized religious forms of spirituality from individualized, non-institutional modes of spiritual practice. A third category, syncretic spirituality, refers to configurations in which elements of institutional religion are combined with NFSP, resulting in hybrid belief systems that integrate both traditional and individualized spiritual orientations. Against this conceptual background, the following section outlines how contemporary young adults increasingly construct individualized spiritual profiles, often combining institutional religion with nonreligious forms of spiritual practice. In many regions of the world, including Europe and North America, traditional religious frameworks—once stable sources of social cohesion and value-based orientation—have weakened. A clear trend toward secularization is evident, particularly within Christian denominations (12–15). At the same time, alternative forms of spirituality have gained importance, especially among individuals aged 18–25. These wide range of non-institutional spiritual activities are promoting through social media, the wellness industry, and online influencers. Although such belief systems are not new - having accompanied humanity for centuries - they have often been described as occult, pagan, or rooted in ancient faith traditions. In recent years, their popularity has grown, accompanied by efforts to rediscover and reframe them (16). This trend also includes the combination of sometimes contradictory belief systems. Some individuals identify as believers in the Christian God while simultaneously engaging in esoteric practices. In other cases, dogmas of institutional religion are rejected, liberalized, or modified to suit individual needs. This phenomenon, known in sociology as the “privatization of belief,” was first described by Thomas Luckmann and reflects a pursuit of personal spiritual agency rather than passive adherence to institutional norms (17). Although practices such as astrology are often perceived as harmless or recreational, studies suggest that they may encourage intuitive information processing, magical thinking, and skepticism toward evidence-based knowledge (18, 19).

### 1.3 Psychological mechanisms linking spirituality and conspiracy thinking

The growing popularity of NFSP among young adults appears to be related to the fulfillment of psychological needs that were once met through institutional religion. Individualized practices centered on intuition, inner energy, or a sense of universal connectedness may enhance subjective well-being but at the same time foster acceptance of empirically unverifiable claims (20, 21). Research indicates that individuals engaged in alternative spiritual practices tend to display specific cognitive-emotional patterns, including a preference for symbolic interpretation, intuitive over analytical reasoning, external locus of control, and greater tolerance for ambiguity—all of which may increase susceptibility to conspiracy thinking (22). These mechanisms promote the interpretation of complex social phenomena through simplified narratives that attribute hidden intentions to covert groups or individuals. This overlap becomes particularly evident when examining the most common types of conspiracy theories, which include political manipulation, information suppression by the medical or pharmaceutical industries, scientific denialism, and spiritual-cosmic conspiracies referring to metaphysical forces. The latter category is especially characteristic of communities associated with NFSP, as it merges metaphysical elements with antagonistic explanations of global events. In contrast, institutional religion can provide coherent worldviews and socially stabilizing frameworks that help buffer uncertainty. However, this relationship is not straightforward: rigid or fundamentalist forms of religiosity may strengthen certain conspiratorial narratives, whereas more moderate, community-oriented religiosity may reduce them (23). These distinctions suggest that different spiritual orientations activate distinct psychological mechanisms—some protective against conspiracy thinking, others increasing vulnerability—shaping how young adults interpret ambiguous or threatening situations.

### 1.4 Young adults as a group particularly vulnerable to environmental influences

Young adults represent a population with heightened susceptibility to both conspiracy beliefs and rapidly changing spiritual paradigms. Emotional instability, identity exploration, and exposure to digital environments saturated with misinformation foster the formation of alternative belief systems (24, 25). In this group, conspiracy beliefs are associated with increased distrust, risky health behaviors, and reduced adherence to medical recommendations (26, 27). Identifying factors that either increase or decrease this susceptibility is therefore of significant importance for preventive psychiatry and mental health promotion. Despite growing interest in conspiracy beliefs and NFSP, existing research has generally examined these domains separately. Few studies have analyzed them jointly or explored the sociodemographic correlates of different spiritual profiles. Even fewer have included individuals with “hybrid” belief systems—those who identify as religious yet engage in other spiritual practices. This gap is particularly relevant in societies undergoing rapid sociocultural change, where systems of belief are becoming increasingly fluid, individualized, and syncretic.

### 1.5 The Polish sociocultural context

Poland provides a particularly interesting context for analyzing these relationships. The country is characterized by a strong Catholic identity, attachment to tradition, high levels of participation in religious rituals, and deeply rooted cultural-religious practices, as well as relative monocultural homogeneity. However, over the past three decades, significant transformations in the worldview structure of the Polish population have taken place. Recent studies clearly indicate a marked decline in institutional religiosity and in religious practice among believers (28).

These shifts may reflect broader social phenomena, including geopolitical instability, declining trust in religious institutions, public debates regarding the social role of the Church, and the growing influence of online misinformation. Despite these transformations, empirical studies examining spirituality and conspiracy beliefs among young adults in Poland remain virtually absent from medical and psychological databases.

Based on a literature review, the following hypotheses were formulated:

1. Higher levels of spirituality are associated with stronger conspiracy thinking among young adults.
2. Individuals engaging in NFSP practices exhibit higher levels of conspiracy thinking than those who do not.
3. Catholic individuals who engage in NFSP practices show stronger conspiracy thinking than Catholics who do not.

## 2 Aim of the study

The aim of the present study was to examine the relationship between the level of spirituality (overall and across its specific domains) and the intensity of conspiracy thinking among young adults in Poland. The analysis also explored whether the type of spirituality—religious, non-religious, or syncretic—differentiated susceptibility to conspiracy thinking.

## 3 Material

The study was conducted between January 2024 and January 2025. Exclusion criteria included ongoing psychiatric or psychological treatment and age below 18 or above 25 years. For surveys conducted in secondary schools, permission was obtained in advance from school administrations. Only questionnaires with complete data on the key variables (spirituality and conspiratorial beliefs) were included in the final analyses. Cases with missing responses on these variables were excluded using listwise deletion. The proportion of excluded cases due to incomplete data was low and did not affect the overall structure of the sample (<3%). Due to the small number of participants, non-binary individuals (n = 10) and those identifying with Orthodox Christianity (1), Jehovah’s Witnesses (4), Protestantism (2), Buddhism (3), and Islam (1) were excluded from the analysis. The final sample comprised 1,100 participants.

Based on participants’ self-reported data, the sample was divided into four groups:

Group A: Catholics (n = 399)

Group B: “Catholics Plus” (Religious + NFSP group = participants identifying as Catholics who also reported engaging in at least one Nonreligious Form of Spiritual Practice; n = 304)

Group C: Atheists (n = 197)

Group D: “Atheists Plus” (participants identifying as atheists who reported engaging in NFSP; n = 200)

## 4 Methods

The following research instruments were used:

### 4.1 Author’s sociodemographic questionnaire

This questionnaire was divided into two sections, A and B. Section A included questions on gender, age, size of hometown, education level, relationship status, and parenthood. Section B contained questions regarding religious affiliation, participation in religious practices (e.g., attending services), upbringing in a religious tradition, and engagement in other spiritual practices. To capture individualized spiritual engagement outside institutional religion, participants were asked about their involvement in Nonreligious Forms of Spiritual Practice (NFSP). For the purpose of this study, NFSP were defined as spiritual practices pursued independently of formal religious traditions and oriented toward personal meaning-making, subjective experience, or metaphysical interpretations of reality. Engagement in non-religious forms of spiritual practice was assessed using a structured set of survey items referring to specific practices commonly described in the literature as individualized or alternative forms of spirituality (e.g., astrology, tarot, crystal therapy, affirmation, meditation, numerology, palo Santo rituals). For each practice, participants were asked three dichotomous (Yes/No) questions addressing different levels of involvement: (1) whether they practiced the given activity, (2) whether they believed in its effectiveness or validity, and (3) whether they used it as a source of guidance in everyday life or decision-making. Participants were allowed to endorse more than one practice. In addition, an open-ended question enabled respondents to report other spiritual practices not listed in the questionnaire. These responses were subsequently reviewed and categorized when they met the operational criteria of NFSP, defined as spiritual activities practiced outside institutional religious contexts and based primarily on individualized belief systems rather than formal doctrine or community affiliation. For analytical purposes, participants were classified as engaging in NFSP if they endorsed at least one practice at any of the three levels (practice, belief, or guidance).

### 4.2 Interfaith spirituality scale by Kira et al., in the Polish adaptation by Surzykiewicz et al.

The ISS consists of 22 items rated on a 4-point Likert scale (1 = definitely not, 4 = definitely yes) (29, 30). The Polish version of the scale has demonstrated high internal consistency (α = 0.96). In this instrument, the Creator is understood broadly—not only as God but also as a higher being or universal force. The scale assesses spirituality across four domains: (1) Direct connection with the Creator (DLC) - a subjective sense of closeness and relationship with the creative force, accompanied by inner peace, the pursuit of transcendence, and a desire to approach this source. (2) Asceticism (self-discipline and virtues ASTC) - includes self-control, moderation, humility, contentment, and the cultivation of virtues such as gratitude, honesty, and kindness. (3) Meditation and Spiritual Knowledge (MED) – reflects the practice of meditation and contemplation, as well as the pursuit of understanding one’s existence and the meaning of life. (4) Spiritual Love (Divine Love DL) – refers to the feeling of love directed toward the Creator and the experience of divine love expressed through relationships with others. The authors describe this domain as the core of spirituality—the ineffable love for the Creator perceived as the essence of spiritual life.

Generic Conspiracist Beliefs Scale (GCBS) by Brotherton, French, and Pickering, in the Polish adaptation by Siwiak et al. (31, 32).

The GCBS comprises 15 items rated on a 5-point Likert scale. It measures the overall tendency to endorse conspiracy beliefs as well as five subdimensions: (1) government malfeasance (criminal activity by government organizations GCBS GM), (2) malevolent global conspiracies (secret groups exerting control over global events GCBS MGC), (3) extraterrestrial cover-up (concealment of contact with extraterrestrial civilizations GCBS ECU), (4) personal well-being (conspiracies related to health and freedom GCBS PW), and (5) control of information (unethical manipulation of information by global organizations GCBS Col). The Polish adaptation demonstrated high internal consistency (Cronbach’s α = 0.93), with subscale reliabilities ranging from 0.73 to 0.89. In the present study, Cronbach’s α for the total scale was 0.94.

### 4.3 Statistical analysis

Data were analyzed using Microsoft Excel and Statistica version 13.3. Normality of variable distribution was assessed using the Kolmogorov–Smirnov test with Lilliefors correction. Because most variables deviated from a normal distribution, nonparametric statistical methods were applied. Differences between independent variables were examined using the Kruskal–Wallis test, followed by Dunn’s *post hoc* test. Associations between continuous variables were analyzed using Spearman’s rank correlation coefficient. The significance level was set at α ≤ 0.05.

### 4.4 Ethics

The study was approved by the Bioethics Committee (No. BNW/NWN/0052/KB/285/23). All procedures were conducted in accordance with the Declaration of Helsinki and the internal ethical guidelines of the hosting institution. Participants were informed about the purpose of the study, the course of procedures, and their right to withdraw at any stage. Informed consent was obtained from all participants prior to inclusion. Data were collected and processed in a manner ensuring anonymity and confidentiality.

## 5 Results

### 5.1 Description of the study group

The sociodemographic characteristics of the study sample were presented in **Table 1**.

**Table 1:** Sociodemographic characteristics of the study group (n = 1,100).

Variable	Total participants	Group 1 (Catholics)	Group 2 (“Catholics plus”)	Group 3 (“Atheists”)	Group 4 (“Atheists plus”)
N	1100	399	304	197	200
Age, Mean ± SD	21.40 ± 2.14	21.37 ± 2.14	21.41 ±2.01	21.36 ±2.20	21.19 ±2.31
Women, n (%)	718 (65.27%)	208 (52.13%)	249 (81.91%)	102 (51.77%)	162 (82.23%)
Cities >100k inhabitants n(%)	562 (51.1%)	195 (48.87%)	136 (44.73%)	110 (55.84%)	116 (58%)
In relationship n(%)	500 (45.45%)	162 (40.6%)	165 (54.27%)	100 (50.76%)	119 (59.5%)
Having children n(%)	18 (1.64%)	10 (2.5%)	6 (1.97%)	0	2 (1%)
Participation in religious rituals n(%)	351/703 (49.92%)	234 (58.64%)	167 (54.93%)		
Affiliation with the Catholic Church, n (%)	722 (65.63%)				
Practice of tarot n (%)	124 (11.27%)		44 (14.5%)		80 (40.0%)
Practice of numerology n (%)	134 (12.18%)		67 (22.0%)		67 (33.5%)
Practice of astrology n (%)	261 (23.73%)		151 (49.67%)		110 (55.0%)
Practice of crystal therapy n (%)	120 (10.9%)		43 (14.14%)		77 (38.5%)
Practice of manifestation/affirmation n (%)	350 (31.81%)		211 (69.41%)		139 (69.5%)
Practice of meditation n (%)	297 (27.0%)		164 (53.95%)		133 (66.5%)
Practice of palo Santo rituals n (%)	55 (5.0%)		20 (6.58%)		35 (17.5%)
Practice of at least two esoteric practices, n(%).	307 (27.90%)		190 (62.5%)		117 (58.5%)

### 5.2 Interfaith spirituality scale

Group 1 achieved the following mean scores (± standard deviation): Spirituality Total = 11.68 ± 2.45; DRC (Direct Relationship with the Creator) = 21.22 ± 7.21; ASTC (Asceticism) = 16.14 ± 2.88; MED (Meditation and Spiritual Knowledge) = 16.51 ± 4.13; DL (Divine Love) = 9.34 ± 2.12.

Group 2 obtained the following results: Spirituality Total = 12.10 ± 2.11; DRC = 21.99 ± 6.03; ASTC = 16.45 ± 2.24; MED = 17.47 ± 3.55; DL = 9.65 ± 1.72.

Group 3 scored as follows: Spirituality Total = 8.14 ± 2.14; DRC = 9.91 ± 3.88; ASTC = 13.18 ± 2.90; MED = 13.15 ± 3.67; DL = 5.81 ± 1.60.

Group 4 obtained: Spirituality Total = 9.93 ± 2.58; DRC = 14.31 ± 6.72; ASTC = 14.28 ± 2.90; MED = 15.46 ± 4.11; DL = 7.39 ± 2.08.

Groups 1 and 2 achieved higher scores in the overall Spirituality Total and across all four domains (DRC, ASTC, MED, DL) compared with Groups 3 and 4. Group 4 scored higher than Group 3 in all domains of spirituality, whereas Group 3 recorded the lowest results on the IFS scale. The differences between the groups were statistically significant (p ≤ 0.05).

### 5.3 General conspiracist beliefs scale

Group 1 achieved the following results (mean ± SD): GCBS Total = 36.09 ± 13.53; GCBS GM (Government Malfeasance) = 7.86 ± 3.41; GCBS MGC (Malevolent Global Conspiracies) = 7.24 ± 3.35; GCBS ECU (Extraterrestrial Cover-Up) = 5.22 ± 2.78; GCBS PW (Personal Well-being) = 7.11 ± 3.17; GCBS Col (Control of Information) = 8.65 ± 3.17.

Group 2 obtained: GCBS Total = 41.47 ± 13.57; GCBS GM = 8.82 ± 3.30; GCBS MGC = 8.24 ± 3.30; GCBS ECU = 6.36 ± 3.22; GCBS PW = 8.29 ± 3.36; GCBS Col = 9.76 ± 2.91.

Group 3 achieved: GCBS Total = 33.18 ± 14.43; GCBS GM = 7.79 ± 3.86; GCBS MGC = 6.21 ± 3.38; GCBS ECU = 4.74 ± 2.88; GCBS PW = 6.15 ± 3.27; GCBS Col = 8.39 ± 3.33.

Group 4 obtained: GCBS Total = 38.14 ± 13.60; GCBS GM = 9.00 ± 3.29; GCBS MGC = 7.26 ± 3.38; GCBS ECU = 5.95 ± 3.29; GCBS PW = 7.06 ± 3.27; GCBS Col = 8.87 ± 3.09.

**Figure 1:**
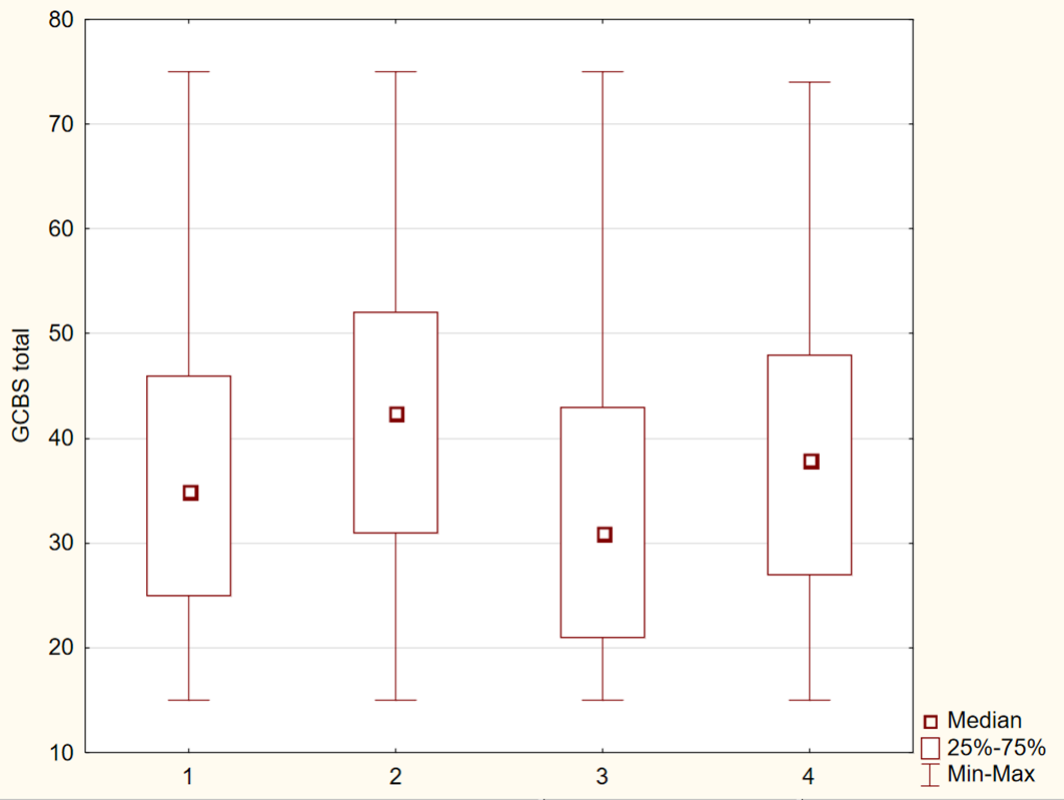
Distribution of GCBS-total scores across four groups.

**Figure 2:**
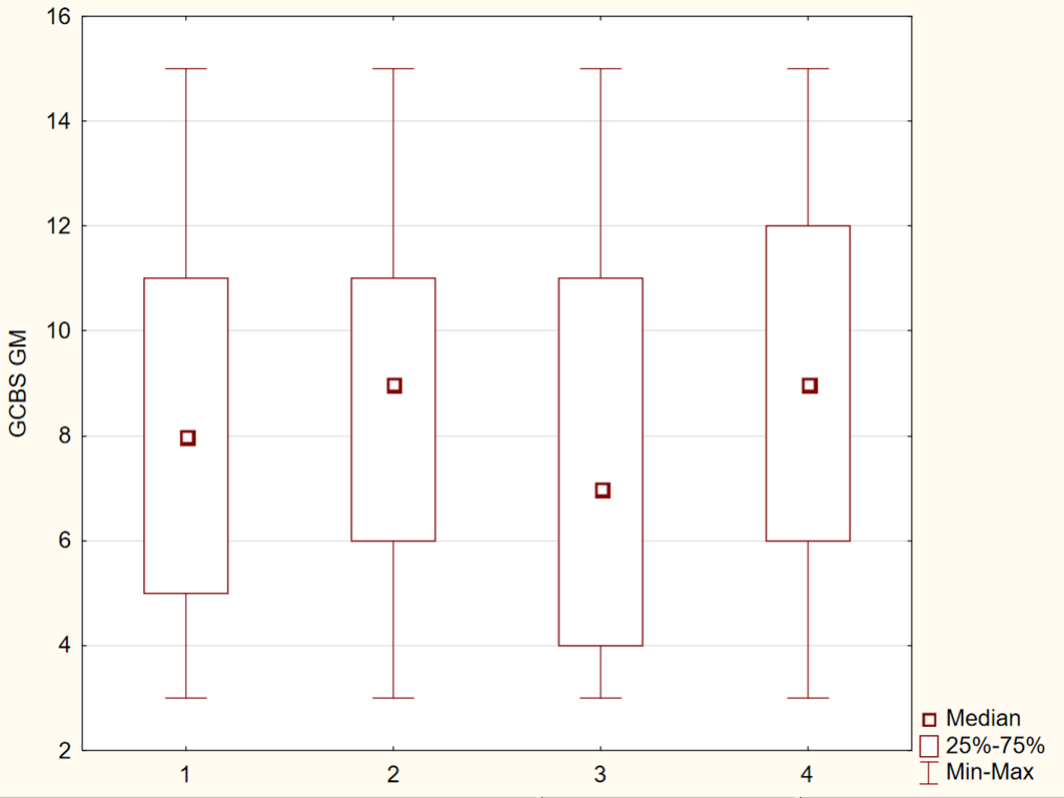
Distribution of GCBS–GM subscale scores across four groups.

**Figure 3:**
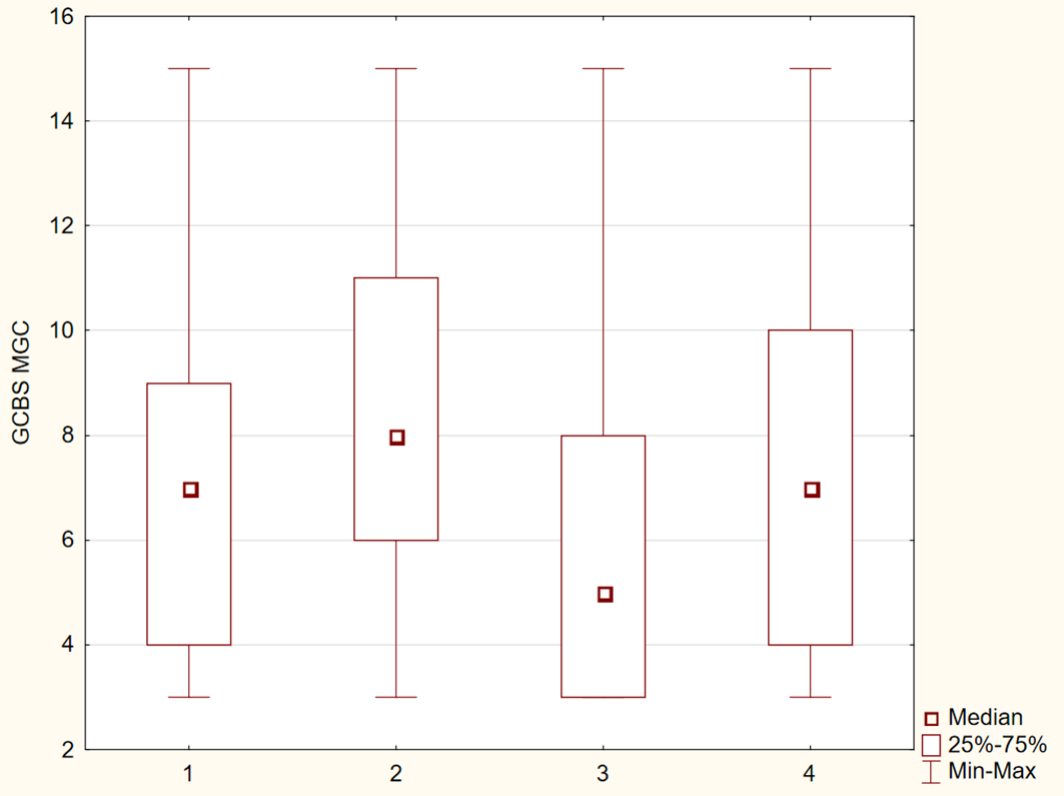
Distribution of GCBS–MGC subscale scores across four groups.

**Figure 4:**
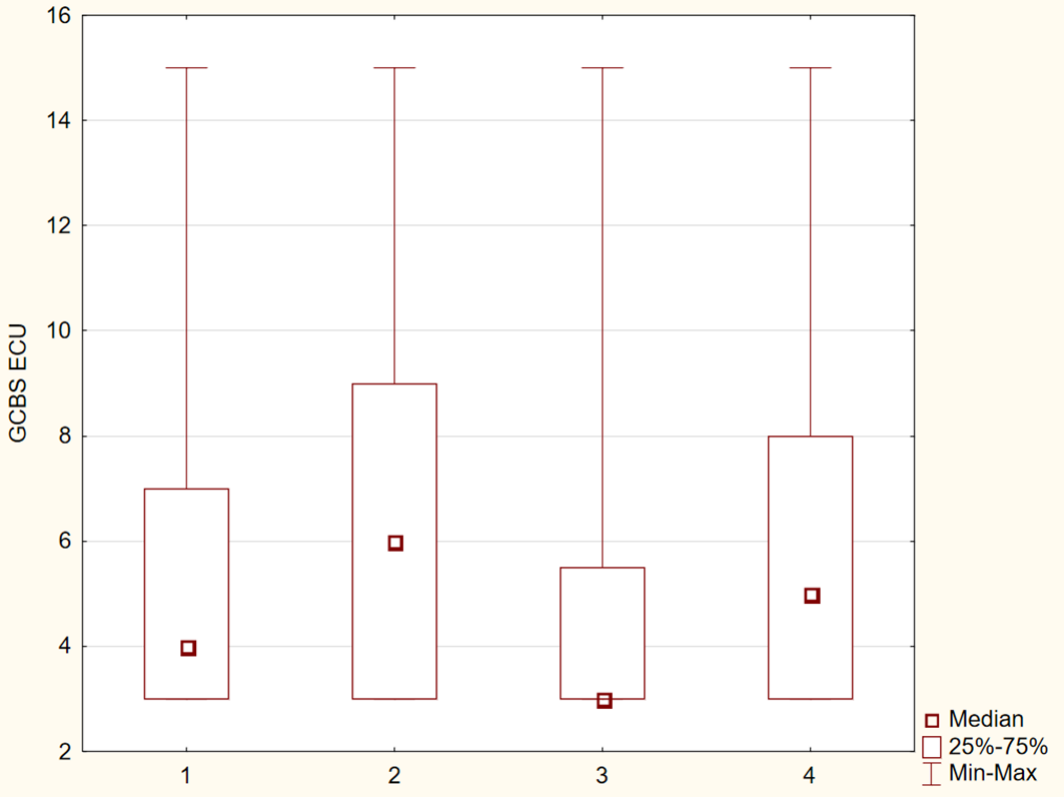
Distribution of GCBS–ECU subscale scores across four groups.

**Figure 5:**
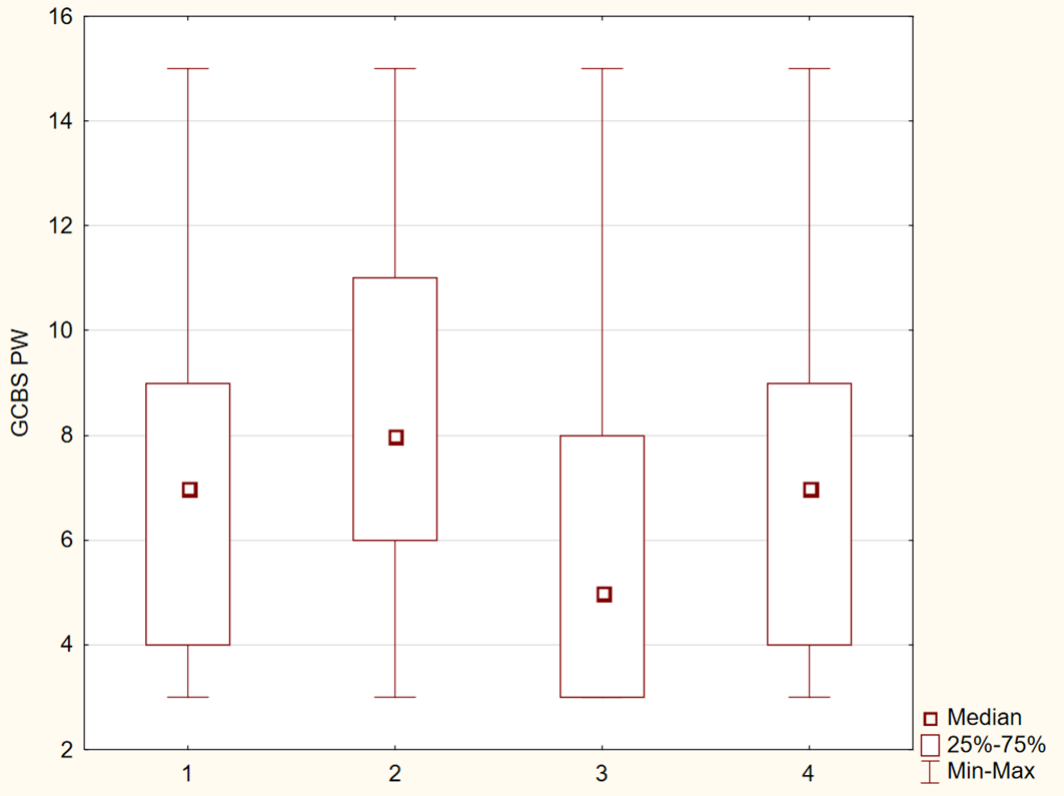
Distribution of GCBS–PW subscale scores across four groups.

**Figure 6:**
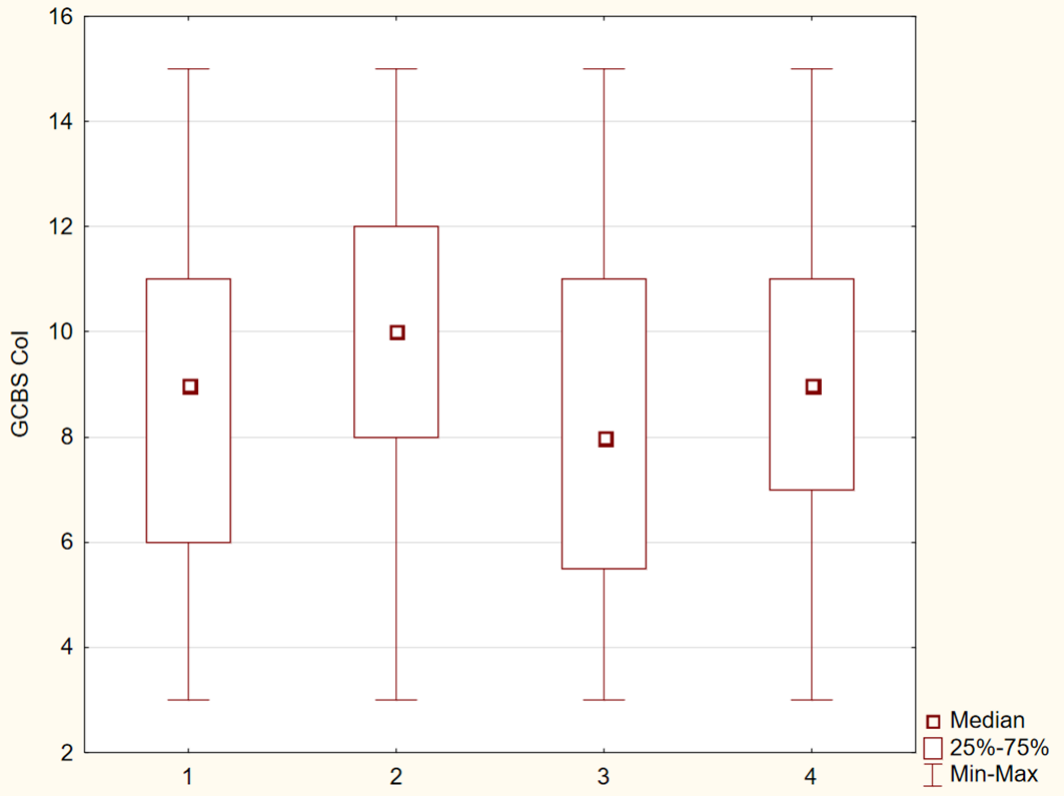
Distribution of GCBS–Col subscale scores across four groups.

The differences between the study groups were statistically significant (p ≤ 0.05). The results were presented in **Figures 1**–**6**.

### 5.4 Correlations between the studied variables

Group 1 (Catholics)

Scores on Spirituality Total were negatively correlated with the GCBS ECU domain (p = 0.033). The Asceticism (ASTC) domain correlated negatively with GCBS Total (p = 0.014) and GCBS ECU (p < 0.001).

Group 2 (Catholics Plus)

Spirituality Total scores correlated positively with the GCBS Col domain (p = 0.04). Scores on the Meditation (MED) domain correlated positively with GCBS Total (p = 0.004), GCBS GM (p = 0.006), GCBS MGC (p = 0.016), GCBS PW (p = 0.02), and GCBS Col (p = 0.002). Scores on the Asceticism (ASTC) domain correlated negatively with GCBS ECU (p = 0.002).

Group 3 (Atheists)

The Asceticism (ASTC) domain correlated negatively with all GCBS domains (p < 0.001). The Direct Relationship with the Creator (DRC) domain correlated positively with GCBS GM (p = 0.036) and GCBS ECU (p = 0.042).

Group 4 (Atheists Plus)

Spirituality Total scores correlated positively with GCBS Total (p = 0.002), GCBS GM (p = 0.003), GCBS MGC (p < 0.001), GCBS ECU (p = 0.038), GCBS PW (p = 0.012), and GCBS Col (p = 0.04). The Direct Relationship with the Creator (DRC) domain correlated positively with all GCBS domains (p < 0.001). The Meditation (MED) domain correlated positively with GCBS Total (p = 0.003), GCBS GM (p = 0.001), GCBS MGC (p = 0.003), and GCBS PW (p = 0.014).

The results were presented in **Table 2**.

**Table 2:** Correlations between spirituality domains (ISS) and General Conspirational Beliefs Scale domains (GCBS).

Variable	GCBS total	Government malfeasance(GCBS GM)	Malevolent global conspiracies (GCBS MGC)	Extraterrestrial cover-up (GCBS ECU)	Personal well-being (GCBS PW)	Control of information(GCBS COL)
Spirituality Total - Group 1	-0.01	-0.03	0.03	-0.1*	0.01	0.03
Spirituality Total - Group 2	0.09	0.05	0.10	0.03	0.09	0.11*
Spirituality Total - Group 4	0.2*	0.2*	0.2*	0.14*	0.17*	0.14*
Direct connections with the Creator (DRC) - Group 3	0.15	0.14*	0.09	0.14*	0.07	0.04
Direct connections with the Creator (DRC) - Group 4	0.25*	0.25*	0.26*	0.17*	0.17*	0.18*
Asceticism (ASTC) - Group 1	-0.11*	-0.06	-0.06	-0.23*	-0.07	-0.08
Asceticism (ASTC) - Group 2	-0.08	-0.07	-0.03	-0.17*	-0.07	-0.01
Asceticism (ASTC) - Group 3	-0.3*	-0.3*	-0.25*	-0.23*	-0.33*	-0.25*
Meditation (MED) - Group 2	0.15*	0.14*	0.13*	0.08	0.12*	0.17*
Meditation (MED) - Group 4	0.19*	0.22*	0.2*	0.09	0.16*	0.13

* - p≤0.005.

Values significant at *p* ≤ .005 are indicated by an asterisk (*) and boldface.

## 6 Discussion

### 6.1 Summary of key empirical findings

The present study revealed three central empirical patterns regarding the relationship between spirituality and conspiratorial thinking among Polish young adults. First, engagement in Nonreligious Forms of Spiritual Practice (NFSP)—both among religious and non-religious participants—was associated with higher levels of conspiratorial thinking across several domains of the GCBS. Second, religious spirituality oriented toward asceticism and moral self-regulation showed a consistent negative association with conspiratorial beliefs, particularly among Catholics. Third, atheists displayed the lowest overall levels of conspiracy thinking; however, atheists who engaged in NFSP exhibited significantly higher scores than atheists without such practices. Taken together, these findings indicate that spirituality is not a unitary construct in relation to conspiratorial thinking. Rather, the form and epistemic orientation of spirituality appear to be decisive, with different spiritual profiles corresponding to distinct patterns of meaning-making and information processing.

### 6.2 Differentiated patterns of spirituality and conspiratorial thinking

The relationship between spirituality and conspiratorial thinking observed in this study is clearly non-linear. Higher spirituality does not uniformly predict either increased or decreased susceptibility to conspiracy beliefs. Instead, the results suggest that institutionally embedded, norm-regulated spirituality and individualized, intuition-oriented spirituality are associated with qualitatively different epistemic styles. In our sample, spirituality grounded in institutional religion - particularly in its ascetic and virtue-oriented dimensions - was associated with lower levels of conspiratorial thinking. By contrast, spirituality expressed through NFSP and syncretic configurations was linked to higher endorsement of conspiracy beliefs. This pattern supports theoretical perspectives that emphasize the role of cognitive style in conspiratorial ideation. Analytic–reflective processing is associated with greater resistance to conspiratorial narratives, whereas intuitive–impressionistic processing increases susceptibility to unverifiable explanations (33–35). Importantly, these findings should be interpreted strictly in associational terms. Given the cross-sectional design of the study, no causal inferences can be drawn. Rather, the data highlight stable co-occurring patterns between spiritual orientation and epistemic attitudes toward knowledge, authority, and hidden explanations.

### 6.3 Religious spirituality as a stabilizing cognitive correlate

Among Catholics, higher scores in the asceticism and moral self-regulation domain were consistently associated with lower conspiratorial thinking, particularly in relation to extraterrestrial cover-up beliefs. This pattern suggests that religious spirituality embedded in institutional frameworks may function as a stabilizing cognitive correlate, supporting disciplined reflection and normative evaluation of information. Such findings align with previous research indicating that religiosity is not uniformly associated with conspiracy beliefs, but that its effects depend on the form of religious engagement and the nature of one’s relationship to doctrine (1, 2, 36). Community-based religiosity grounded in shared norms and moral regulation may provide coherent interpretative schemas that reduce the appeal of alternative, conspiratorial explanations in situations of uncertainty. It is important to emphasize that the present study did not assess religious fundamentalism, doctrinal rigidity, or literalist belief styles. Therefore, our interpretation is restricted to the forms of religiosity represented in this sample, which appear to reflect culturally embedded and predominantly moderate Catholic identity.

### 6.4 Syncretic spirituality: religious identity combined with NFSP

Participants who combined Catholic identity with NFSP represent a syncretic spiritual profile characterized by the coexistence of institutional affiliation and individualized spiritual practice. Empirically, this group exhibited the highest levels of conspiratorial thinking across domains. Rather than interpreting this pattern in theological terms, we conceptualize it as an expression of privatized religiosity (17, 37), in which religious identity is maintained primarily at the declarative level, while meaning-making increasingly relies on personal intuition and non-institutional sources of authority. Such configurations may foster epistemic mistrust toward both religious and scientific institutions, thereby increasing openness to alternative explanatory narratives, including conspiracy theories. This interpretation is consistent with sociological analyses of contemporary spirituality that describe the growing prevalence of hybrid belief systems combining elements of institutional religion with individualized spiritual practices (37, 38). It also resonates with empirical findings suggesting that religious individuals who incorporate alternative spiritual elements are more prone to conspiratorial thinking than those who maintain more coherent institutional frameworks of belief (39, 40).

### 6.5 Atheism, NFSP, and intuitive meaning-making

Atheists without engagement in NFSP displayed the lowest levels of conspiratorial thinking in the sample, a pattern consistent with research linking lower spirituality to a preference for scientific, analytic, and probabilistic explanations (41–43). However, atheists who engaged in NFSP showed markedly higher endorsement of conspiracy beliefs. This contrast suggests that it is not atheism per se that predicts lower conspiratorial thinking, but rather the absence of spiritualized, intuition-based meaning-making. When non-religious individuals adopt individualized spiritual practices, they may become more receptive to epistemic styles emphasizing hidden meanings, personal insight, and alternative sources of truth—features that overlap with conspiratorial worldviews. Within this group, the asceticism domain retained a protective association with conspiratorial thinking, whereas domains reflecting intuitive connection with transcendent forces were positively related to selected conspiracy dimensions. This internal differentiation underscores the heterogeneity of spirituality among atheists and highlights the importance of distinguishing between self-regulatory spirituality and intuition-centered spirituality in psychological research.

### 6.6 Conspirituality as an interpretative framework

The pattern observed among participants engaged in NFSP can be interpreted through the concept of conspirituality. The term was introduced by Ward and Voas to describe the convergence of conspiracy thinking and New Age–type spirituality, reflecting a worldview in which mistrust of institutional authority coexists with beliefs in hidden knowledge and personal spiritual insight (44, 45). This framework emphasizes how individualized spiritual beliefs centered on hidden knowledge, intuition, and personal revelation may coexist with, and reinforce, narratives of concealed power structures and epistemic mistrust. From this perspective, spirituality detached from institutional regulation does not merely represent a private existential orientation but may also shape broader epistemic attitudes. When combined with low trust in social institutions, it can facilitate openness to alternative explanatory systems, including conspiracy theories. Empirical studies consistently show that preference for intuitive reasoning and lower analytic thinking is associated with higher endorsement of conspiratorial and pseudoscientific beliefs (35, 46, 47). Importantly, in the present study, non-institutional spirituality is not equated with esotericism in a normative sense. Rather, it refers descriptively to spiritual practices pursued outside formal religious traditions, which—under certain social and cognitive conditions—may co-occur with heightened susceptibility to conspiratorial narratives.

### 6.7 Clinical and preventive implications

From a clinical and preventive perspective, the findings suggest that spirituality should not be treated as a uniformly protective or risk factor. Instead, its form, institutional embeddedness, and epistemic orientation appear to be crucial. Reflective, norm-regulated spiritual orientations may support cognitive stability and epistemic vigilance, whereas highly individualized, intuition-based spiritual practices may co-occur with heightened epistemic mistrust and greater openness to conspiratorial explanations. These results do not imply that NFSP are inherently maladaptive. However, they point to the importance of considering patients’ spiritual profiles in mental health contexts, particularly with regard to their attitudes toward knowledge, authority, and evidence-based recommendations. For clinicians working with young adults, exploring not only declared religious affiliation but also individualized spiritual practices may provide valuable insight into patients’ meaning-making strategies and potential vulnerabilities to misinformation and conspiracist narratives. At a preventive level, the findings underscore the need for psychoeducational interventions that integrate critical thinking and media literacy with culturally sensitive discussion of spirituality, hidden knowledge, and the limits of epistemic trust—especially in societies undergoing rapid transformation of belief systems.

## 7 Conclusions

Engagement in Nonreligious Forms of Spiritual Practice (NFSP) was consistently associated with higher levels of conspiratorial thinking among Polish young adults, both in religious and non-religious participants.

Religious spirituality oriented toward asceticism and moral self-regulation was negatively associated with conspiratorial beliefs, particularly among Catholics. These associations suggest that institutionally embedded, norm-regulated forms of spirituality may co-occur with greater epistemic stability and lower openness to conspiratorial explanations.

Atheists without engagement in NFSP displayed the lowest overall levels of conspiratorial thinking in the sample. However, atheists who practiced NFSP showed substantially higher endorsement of conspiracy beliefs, indicating that it is not atheism per se, but rather the presence or absence of individualized spiritual practices, that differentiates susceptibility to conspiratorial thinking.

The findings underscore that spirituality is not a homogeneous construct in relation to conspiratorial thinking. Depending on its form—religious, non-religious (NFSP-based), or syncretic—it may be associated with either lower or higher levels of conspiracy beliefs.

From a clinical and preventive perspective, the results suggest that assessing young adults’ spiritual profiles - including engagement in NFSP - may provide valuable context for understanding their epistemic attitudes toward authority, science, and evidence-based recommendations. Such assessments should, however, be used cautiously and without normative assumptions about the adaptiveness or maladaptiveness of specific spiritual practices.

## 8 Study limitations

The findings of the present study reflect patterns observed among young adults in Poland and should be interpreted within this specific sociocultural context. Poland is characterized by a historically strong Catholic tradition alongside rapid processes of secularization and individualization of belief, which may limit the direct generalizability of the results to societies with different religious and cultural profiles. Participants declaring religious affiliations other than Catholicism were excluded from the analyses due to the very small size of these subgroups. Consequently, the present findings cannot be generalized to young adults representing other religious traditions, and future research should examine whether similar patterns emerge in more religiously diverse samples. Women were overrepresented in the sample, whereas sex distribution in the general population is more balanced. Although both female and male subsamples were sufficiently large for statistical analyses, this imbalance may have influenced the overall pattern of results and should be taken into account when interpreting the findings. The cross-sectional design of the study constitutes another important limitation, as it precludes any conclusions regarding the direction of effects or causal relationships between spirituality and conspiratorial thinking. Longitudinal and experimental designs are needed to clarify whether certain forms of spirituality precede changes in conspiratorial beliefs or whether the relationship operates in the opposite direction. With regard to the assessment of spiritual practices, the present study focused on the presence or absence of engagement in Nonreligious Forms of Spiritual Practice (NFSP) rather than on the frequency, intensity, or subjective meaning of these practices. This binary operationalization may have obscured important within-group differences and limited the precision of the analyses. Future studies should adopt more fine-grained measures capturing the diversity of spiritual engagement. The study did not differentiate between subtypes of meditation, which may affect the generalizability of associations observed between meditation-related practices and conspiratorial thinking. Importantly, meditation was not conceptualized as esoteric per se. In the present study, it was classified as NFSP only when practiced outside institutional religious contexts and reported by participants as part of their individualized, non-religious spiritual engagement. This distinction acknowledges the long-standing traditions of Christian, Buddhist, and other forms of religious meditation, while also reflecting the empirical observation that meditation is widely practiced today in secular and individualized spiritual contexts. Another methodological limitation concerns the reliance on self-report measures. Both spirituality and conspiratorial thinking were assessed through questionnaires, which may be affected by social desirability, response styles, and individual differences in self-awareness. Moreover, engagement in NFSP was based on participants’ subjective declarations and was not independently verified. Finally, although questionnaires with missing data on key variables were excluded from the final analyses, the use of listwise deletion may have introduced a degree of selection bias, even if the proportion of excluded cases was small. Future research could benefit from applying advanced methods for handling missing data, such as multiple imputation, to further strengthen the robustness of findings.

## Data Availability

The raw data supporting the conclusions of this article will be made available by the authors, without undue reservation.
